# Coronary microvascular dysfunction in hypertrophic cardiomyopathy detected by Rubidium-82 positron emission tomography and cardiac magnetic resonance imaging

**DOI:** 10.1007/s12350-018-1245-4

**Published:** 2018-03-07

**Authors:** William E. Moody, Matthias Schmitt, Parthiban Arumugam

**Affiliations:** 1 Department of Cardiology, Nuffield House, Queen Elizabeth Hospital Birmingham, Birmingham, B15 2TH UK; 2 Nuclear Medicine Centre, Manchester Royal Infirmary, Manchester, M13 9WL UK; 3 Department of Cardiology, University Hospital of South Manchester NHS Foundation Trust, Southmoor Road, Wythenshawe, Manchester, M23 9LT UK

## Case Report

**Figure 1:**
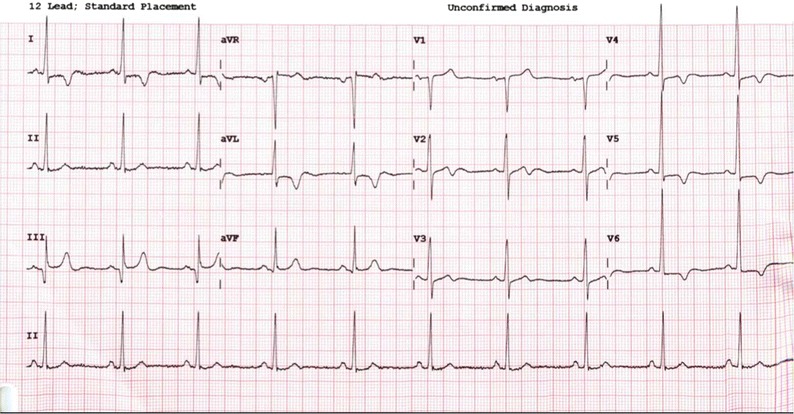
A 12-lead electrocardiogram showed sinus rhythm with T wave inversion in I, aVL, V4–V6 and fulfilled voltage criteria for left ventricular hypertrophy

**Figure 2:**
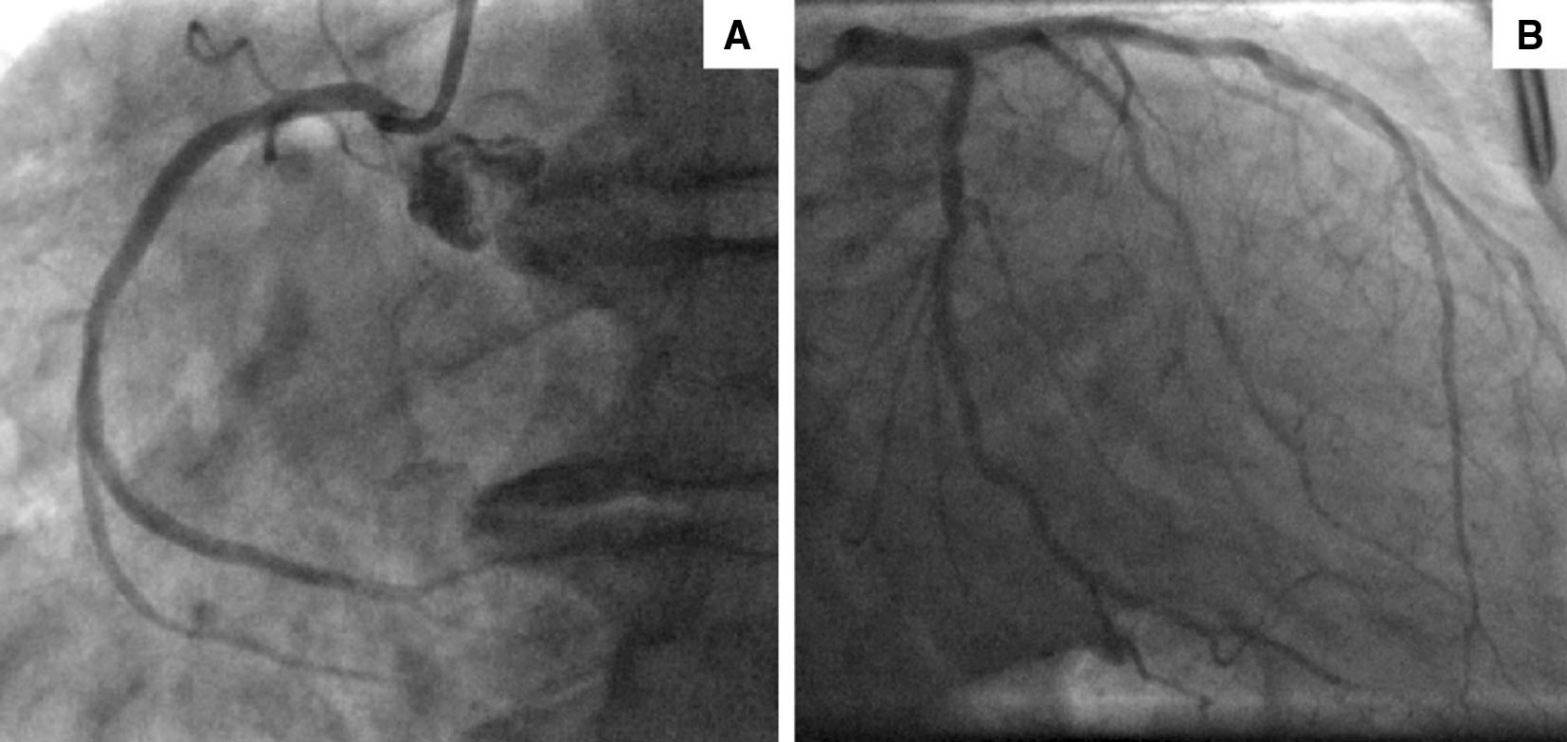
Angiography of the right (**A**) and left (**B**) coronary arteries showing diffuse atheroma with non-flow limiting disease

**Figure 3:**
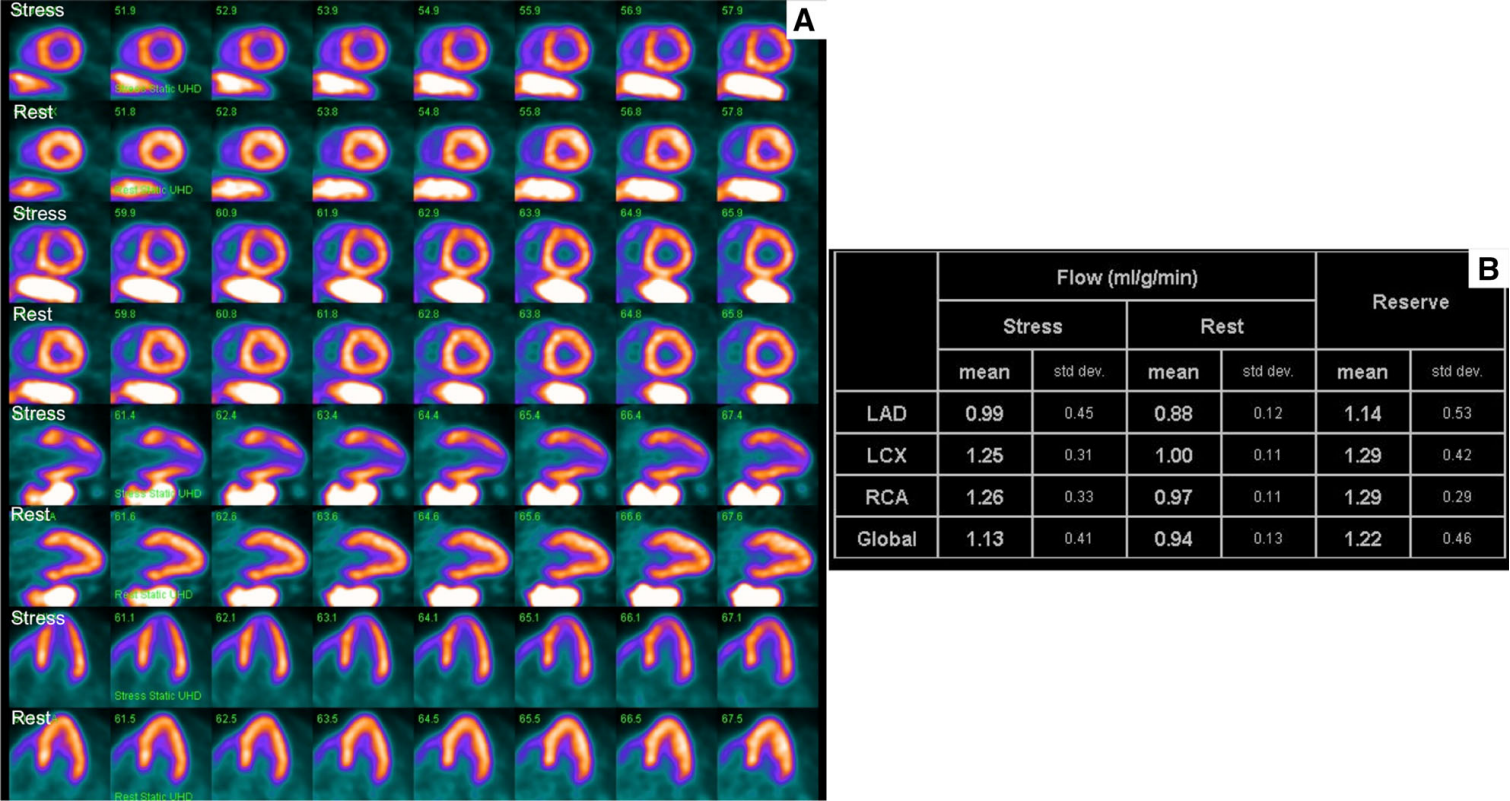
Rubidium-82 positron-emission tomography imaging showed increased tracer uptake at rest, suggestive of left ventricular hypertrophy. Transient ischaemic dilatation was noted with evidence of reversible hypoperfusion in a left anterior descending artery territory (**A**). The maximal global blood flow and the global myocardial perfusion reserve were both reduced (**B**)

**Figure 4:**
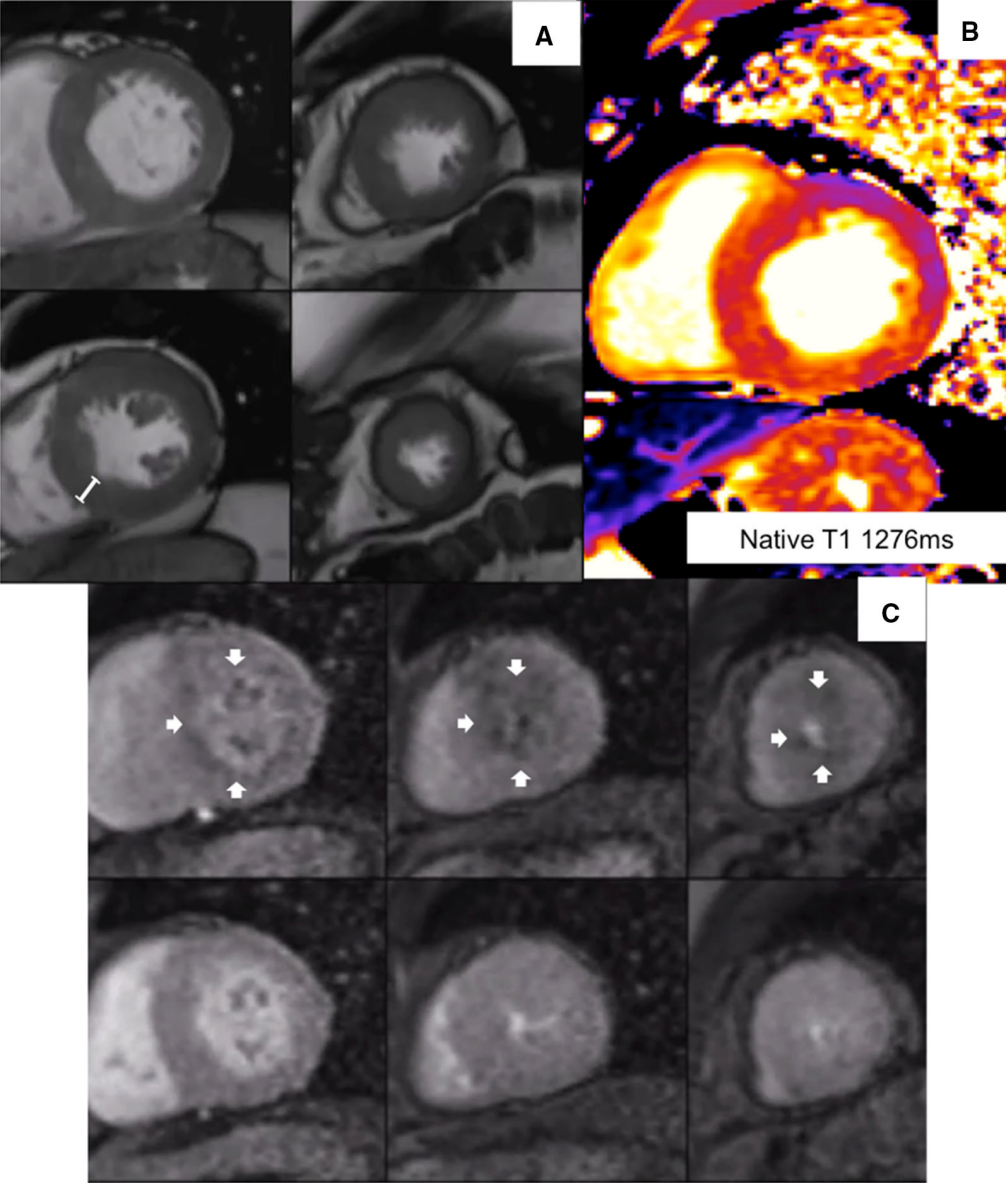
Steady-state free precession cine imaging (**A** and see on-line Video A) demonstrated asymmetric septal hypertrophy with a maximal wall thickness of 19 mm in the mid inferoseptum (see caliper). Left ventricular ejection fraction was supranormal (82%) with apical systolic cavity obliteration. Pre-contrast (native) T1 was increased in the mid septum at 1276 ms consistent with diffuse fibrosis (normal range 1052 ± 23 ms; **B**). Adenosine stress imaging demonstrated a circumferential epicardial–endocardial signal intensity gradient, most pronounced in the basal and mid septal wall—the area of maximal myocardial wall thickness (**C**)

A 75-year-old South-East Asian man presented with exertional chest pain. Risk factors for coronary disease included hypertension, diabetes mellitus, hyperlipidaemia, and family history. High sensitivity Troponin was normal. An electrocardiogram (ECG) showed sinus rhythm with deep T wave inversion in leads I, aVL, V4–V6 (Figure 1). Coronary angiography showed diffuse, non-obstructive disease (Figure 2). Rubidium-82 positron-emission tomography (PET) imaging demonstrated increased tracer uptake at rest, suggestive of left ventricular (LV) hypertrophy. There was adenosine stress-induced LV cavity dilation with reversible hypoperfusion in a left anterior descending artery territory (Figure 3A). The global myocardial perfusion reserve (MPR) was reduced at 1.22 ml/g/min (Figure 3B). In view of the resting ECG abnormality, high tracer uptake at rest and reduced global in the context of non-obstructed coronaries, cardiac magnetic resonance (CMR) imaging (3T Skyra) was performed to exclude a cardiomyopathy. This demonstrated marked regional variability in heart muscle thickness with a maximal wall thickness of 19 mm in the mid inferoseptum (Figure 4A). Left ventricular ejection fraction was supranormal (82%) with apical systolic cavity obliteration (see on-line Video A). Although there was only minimal late gadolinium enhancement seen involving the superior right ventricular insertion point (image not shown), native T1 was elevated at 1276 ms consistent with diffuse fibrosis (mid septum, normal range 1052 ± 23 ms; Figure 4B). Adenosine stress imaging demonstrated a circumferential epicardial-endocardial signal intensity gradient, most pronounced in areas of maximal myocardial thickness (Figure 4C, arrows; on-line Video B).

## Discussion

This case highlights the ability of both PET and CMR imaging to demonstrate impaired myocardial blood flow during vasodilator stress with adenosine in a patient with hypertrophic cardiomyopathy (HCM). In the absence of a coronary stenosis, these findings are consistent with microvascular dysfunction. Numerous pathophysiological factors have been implicated in the development of small vessel ischemia in HCM including thickening of intramural coronary arterioles, elevated regional wall stress associated with increased intra-cavity pressures resulting from outflow obstruction, disorganized cellular architecture with increased collagen deposition in the interstitium, and ventricular hypertrophy associated with a decrease in capillary density.^1^ In a study of 34 patients with HCM who underwent combined PET and CMR imaging, Sotgia et al. demonstrated a continuum of coronary microvascular ischemia ranging from those subjects with only mildly elevated wall thickness in whom hyperemic myocardial blood flow (MBF) is relatively preserved and late gadolinium enhancement absent, to those with greater hypertrophy, where MBF is increasingly impaired and replacement fibrosis more frequent and extensive.^2^ Similarly, pixel-wise quantitative CMR perfusion imaging in 35 HCM patients demonstrated an inverse association between hyperemic MBF and wall thickness, with a significantly lower probability of fibrosis in myocardial segments with increasing hyperemic MBF.^3^ These data provide support for the hypothesis that coronary microvascular dysfunction contributes to the development of myocardial fibrosis in HCM. Although in our case there was limited evidence of replacement fibrosis, T1-mapping was consistent with diffuse fibrosis, and this was associated with reduced hyperemic MBF and global MPR. We also demonstrated transient ischemic dilatation on PET which has been reported in up to half of HCM patients without epicardial coronary disease;^4^ this observation is also associated with reduced maximal global hyperemic MBF and MPR, as well as greater maximal wall thickness on echocardiography.^4^,^5^ More recently, PET imaging of 104 HCM patients demonstrated stress-induced LV cavity dilatation in 52% and this was associated with the stress trans-mural perfusion gradient, reduced global MPR and lower stress LV ejection fraction.^6^

The presence of microvascular dysfunction in patients with HCM has long been recognized as a strong predictor of clinical deterioration and mortality: in 2003, Cecchi et al. used PET to identify a stress MBF of < 1.10 ml/g/min as the optimal cut-off for predicting adverse events.^7^ Further long-term outcome studies are required to clarify this threshold and to determine whether this phenomenon provides incremental prognostic utility beyond the factors already entered into the current HCM risk algorithm.^8^

## Electronic supplementary material

Below is the link to the electronic supplementary material.

Supplementary material 1 (PPTX 6072 kb)
