# Supplementary figures and images for: Coronary microvascular dysfunction in hypertrophic cardiomyopathy detected by Rubidium-82 positron emission tomography and cardiac magnetic resonance imaging
Source: J Nucl Cardiol. 2018 Mar 7;26(2):666–70. doi: 10.1007/s12350-018-1245-4 (PMC6430745; doi:10.1007/s12350-018-1245-4)

## Slide 1
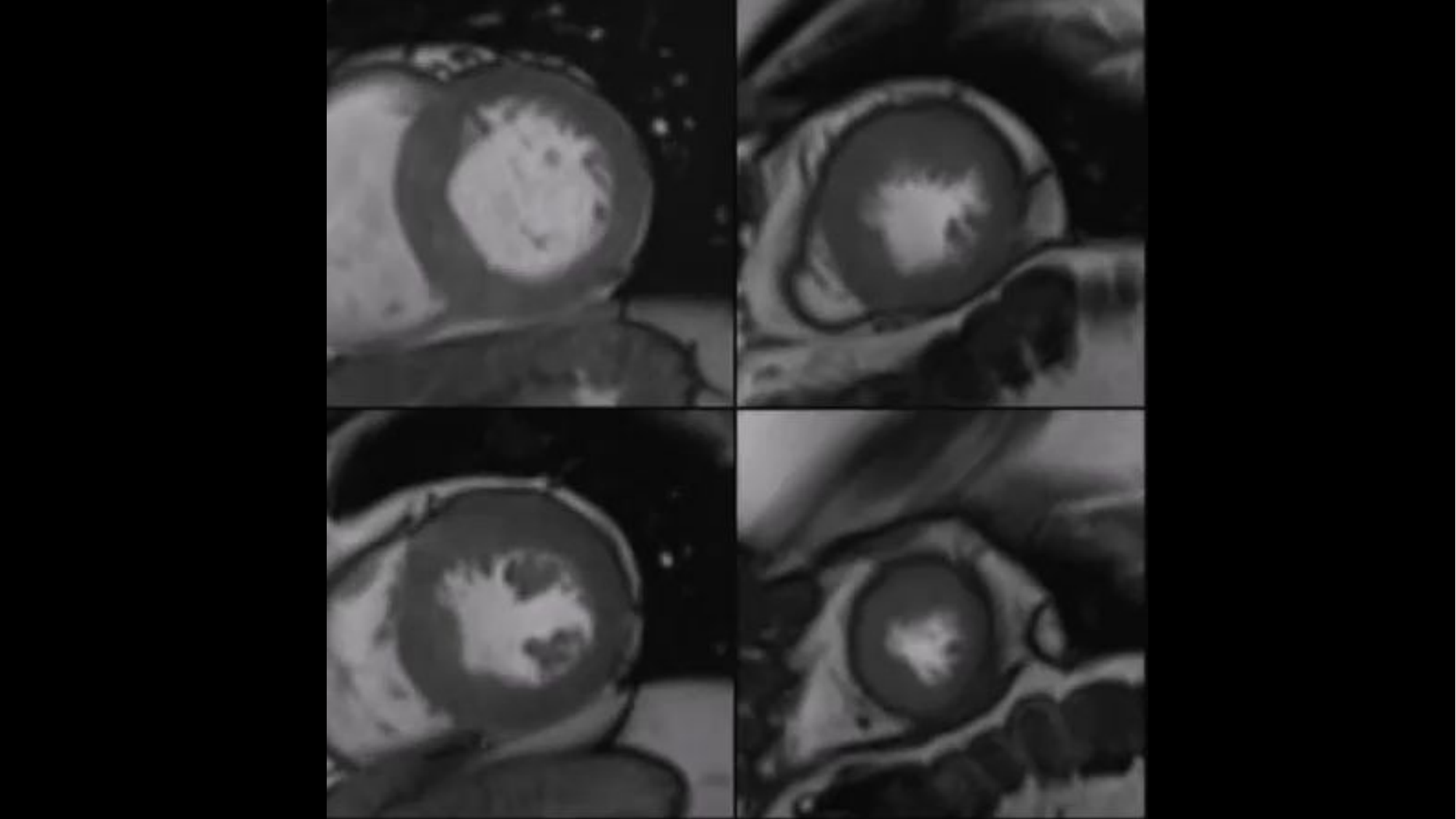

## Slide 2
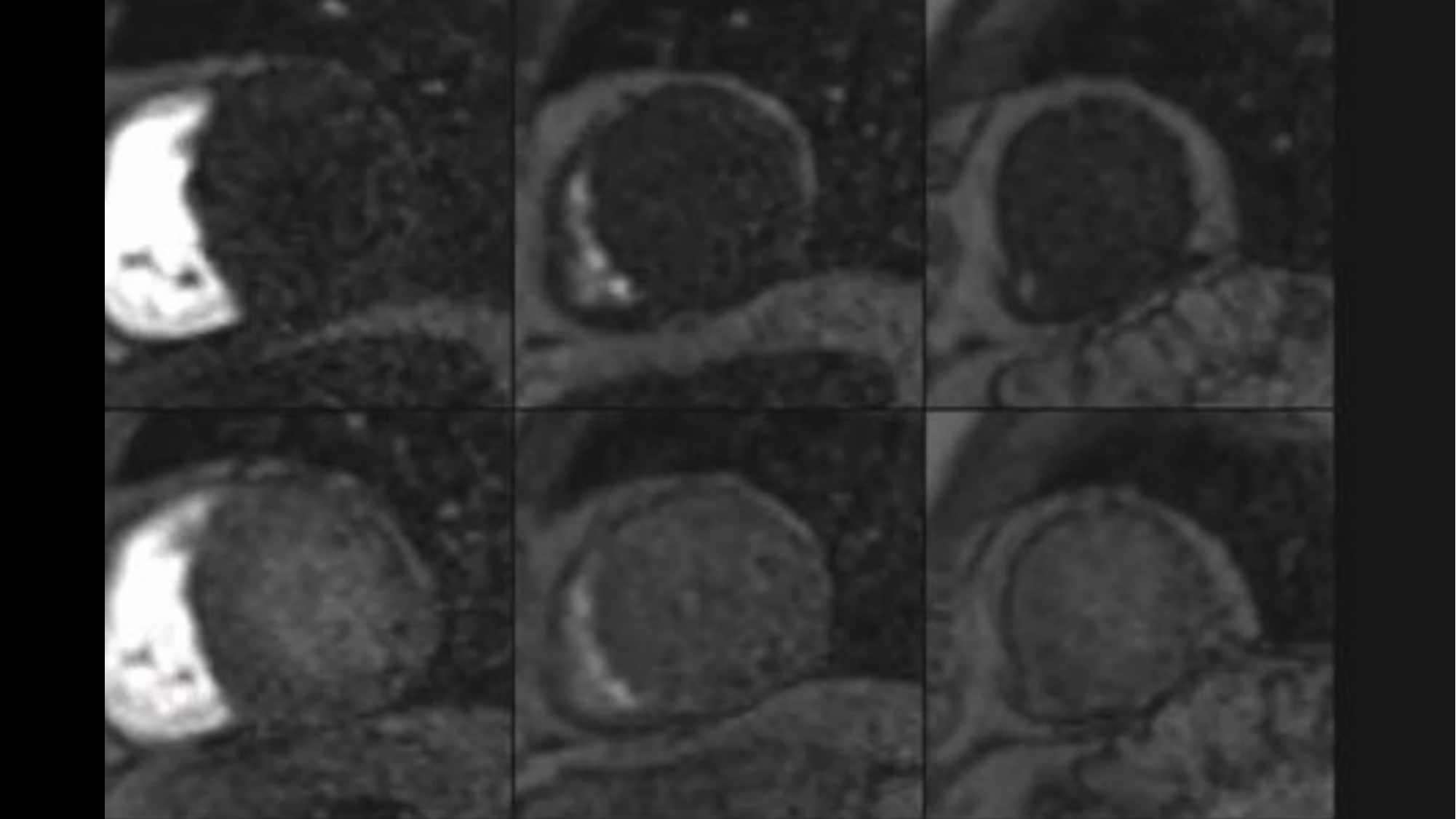

Supplement: Supplementary file 1 — Supplementary material 1 (PPTX 6072 kb) [file 12350_2018_1245_MOESM1_ESM.pptx]
